# Questionnaire design for assessing the pedestrians’ knowledge and attitude toward the visible clothing (case study: Tabriz) 

**Published:** 2019-08

**Authors:** Mirbahador Yazdani, Mahsa Jafari, Mahsa Oustan, Zahra Abedinipour, Saba Mirzahosseini, Negar Amani, Zahra Norouzshenas

**Affiliations:** ^*a*^Road Traffic Injury Research Center, Tabriz, Iran.; ^*b*^School of Civil Engineering, Iran University of Science & Technology, Tehran, Iran.; ^*c*^Faculty of Civil Engineering, University of Tabriz, Tabriz, Iran.

## Abstract

**Background:**

According to the report of World Health Organization (WHO), in 2017, about 20% of the traffic crash fatalities are pedestrians that increases to 50% in Iran. One of the major factors of pedestrians’ crashes at night, is the invisibility of pedestrians at night due to their clothing color.

**Methods:**

In this study, a questionnaire of 25 questions has been designed. These questions were designed in four groups of the crash hazard experience at night, choosing visible clothing, the knowledge of choosing visible clothing and the supply of visible clothing at the living district. The content validity and Cronbachs α (alpha) were implemented for testing the validity and reliability of the study respectively.

**Results:**

The results showed that 3 questions had lower content validity which were rejected and also the content validity and Cronbachs α got the acceptable scores of 0.8 and 0.95, in respect.

**Conclusions:**

According to the used clothing in Iran, this study can reveal the insufficient supply of visible clothing, inadequate use of visible clothing in society and little knowledge of people in this field.

**Keywords:**

Questionnaire, Visibility, Pedestrian, Attitude, Clothing

